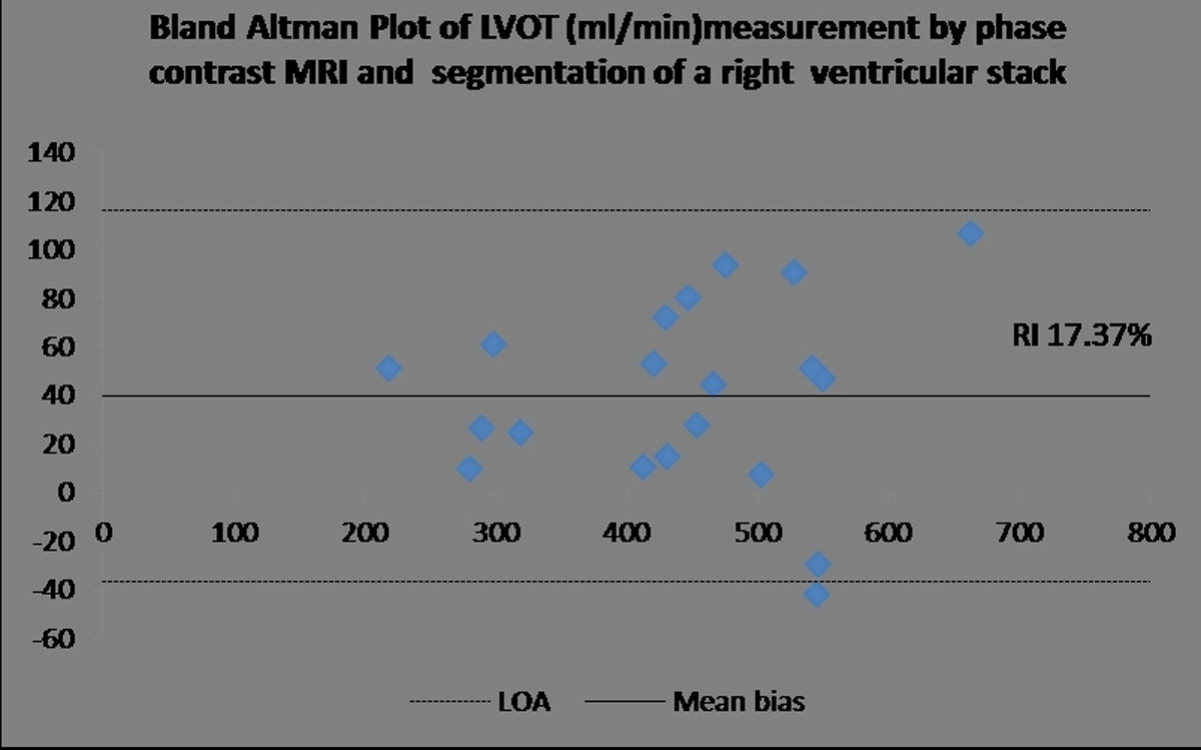# Validation of MRI quantification of biventricular cardiac function in term and preterm neonates

**DOI:** 10.1186/1532-429X-15-S1-P294

**Published:** 2013-01-30

**Authors:** Anna Finnemore, Anthony N Price, Giuliana Durighel, Kathryn Broadhouse, David J Cox, Alan Groves

**Affiliations:** 1 Imaging Sciences, Imperial College, London, UK

## Background

Accurate measurement of neonatal cardiac function remains a key goal to improve understanding of circulatory pathophysiology in prematurity. Cardiac MRI has been shown to be viable in this population, but robust validation is crucial.

Our aim was to assess the accuracy of cardiac functional measures derived from MRI ventricular stack segmentation against phase contrast measures in term and preterm neonates.

## Methods

Cardiac MRI scans were performed on a 3T Philips Achieva scanner (Best, Netherlands) in 40 infants (gestational age 24+3-40+2weeks/ weight at scan 750-3120g). Left ventricular stacks were segmented for epicardial and endocardial contours at end systole and end diastole using Segment software (http://segment.heiberg.se) with manual correction of segmentation where necessary. Correction for long axis motion was performed using the mean of three measures of atrio-ventricular valve descent from a 4 chamber view. Right ventricular stacks were segmented for endocardial contours on 20 infants (gestational age 24+3-40+2weeks/ weight at scan 790-3120g) using the same technique. Cardiac output (CO) was measured by semi-automatic segmentation (View Forum,Philips) of phase contrast(PC) images of the left ventricular outflow tract. The two techniques were compared using a Bland Altman plot. Infants with known patent ductus arteriosus were excluded.

## Results

Bland Altman analysis of left ventricular output by stack and PC showed a mean bias of 14.2 ml/min, with limits of agreement of -54.8 to 83.2 ml/min,, corresponding to a repeatability index (RI, limits of agreement/mean of population) of 16.6%. Bland Altman analysis of right ventricular output by stack and left ventricular output by PC showed a mean bias of 39.7 ml/min, with limits of agreement of -36.9 to 116.5 ml/min corresponding to a repeatability index (RI) of 17.3%. For comparison Bland Altman of neonatal heart rate during by stack and PC imaging showed a mean bias of -2.12 beats/min, with limits of agreement of -19.21 to 14.96 beats/min, corresponding to a repeatability index (RI) of 11.92%.

## Conclusions

Given the wide variability of heart rates and potential for motion in preterm neonatal scanning, our findings suggest stack segmentation at our improved resolution can give relatively accurate measures of preterm cardiac function.

## Funding

Dr Finnemore is a British Heart Foundation Clinical Training Fellow

**Figure 1:**
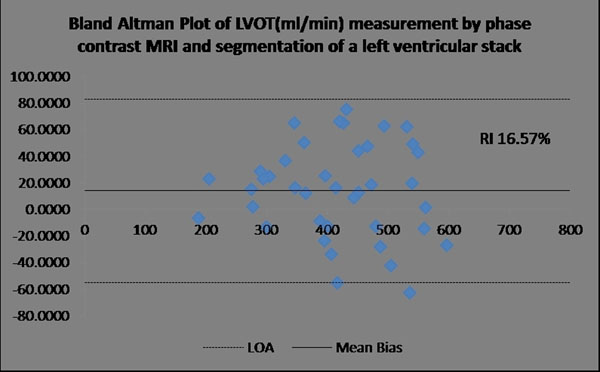


**Figure 2:**